# The role of human papillomavirus genotyping for detecting high-grade intraepithelial neoplasia or cancer in HPV-positive women with normal cytology: a study from a hospital in northeastern China

**DOI:** 10.1186/s12885-020-06935-w

**Published:** 2020-05-19

**Authors:** Jing Zhang, Deyu Zhang, Zhuo Yang, Xiaobin Wang, Danbo Wang

**Affiliations:** 1 Department of Gynecology, Cancer Hospital of China Medical University, Liaoning Cancer Hospital and Institute, No.44 Xiaoheyan Road, Shenyang, 110042 Liaoning province China; 2 Department of Obstetrics and Gynecology, Shengjing Hospital, China Medical University, No.36 Sanhao Street, Shenyang, 110004 Liaoning province China

**Keywords:** Human papillomavirus, hrHPV prevalence, HPV genotyping, High-grade squamous intraepithelial lesion, Cervical cancer

## Abstract

**Background:**

Human papillomavirus (HPV) testing is more sensitive than cytology for detecting cervical cancer and its precursors. This study aimed to analyze the prevalence of high-risk HPV genotypes and evaluate the role of HPV genotyping triage for detecting high-grade squamous intraepithelial lesions, adenocarcinoma in situ and cervical cancer (HSIL+) in HPV-positive women with normal cytology.

**Methods:**

A retrospective study was performed in women who had undergone co-screening at the China Medical University-affiliated Shengjing Hospital between 2012 and 2014.

**Results:**

Of the 34,587 women, 2665 HPV-positive women with normal cytology who had received colposcopy were eligible for analysis. In HSIL+ groups of 204 women, the common genotypes were HPV16, HPV52, HPV58, HPV33, HPV31 and HPV18 in order of prevalence. The proportion of histological HSIL+ in women infected with HPV33 or HPV31 was not significantly different compared to women infected with HPV16 (*P* = 0.30, *P* = 0.19, respectively). The odds ratios for histological HSIL+ were 3.26 (95% confidence interval [CI]: 2.41–4.40) in women with HPV16/18, 4.21 (95% CI: 2.99–5.93) in those with HPV16/18/31/33, and 5.73 (95% CI: 3.30–9.97) in those with HPV16/18/31/33/52/58. Including HPV31/33 genotyping together with HPV16/18 significantly increased the proportion of HSIL+ detection from 63.2 to 77.5% (*P* = 0.002) without significantly increasing the colposcopy per HSIL+ detection ratio (7.7 to 8.1, *P* = 0.66).

**Conclusions:**

HPV genotyping played an important role in managing HPV-positive women with normal cytology. Genotyping for HPV31/33 should be added to the previously recommended HPV16/18 genotyping in triaging HPV-positive women in northeastern China.

## Background

Cervical cancer is the fourth most common cancer among women worldwide and the leading cause of death from cancer in developing countries [1]. Approximately 40 human papillomavirus (HPV) genotypes are associated with infection of the lower genital tract [2]. HPVs are classified as high- or low-risk according to their oncogenic potential [3]. Persistent infection of high-risk HPV (hrHPV) is necessary for developing cervical cancer and its precursors [4].

Recently, HPV genotyping has been accepted in preference to cytology for detecting cervical cancer and its precursors, due to its higher sensitivity [5]. Based on guidelines published in 2012 by the American Cancer Society (ACS), the American Society for Colposcopy and Cervical Pathology (ASCCP), and the American Society for Clinical Pathology (ASCP), a combination of cervical cytology and HPV testing (co-screening) is the preferred screening method for women aged 30–65 years old. It is recommended that HPV16/18-positive women with normal cytology should be referred for immediate colposcopy, whereas those testing positive for other hrHPV genotypes should be followed up in 1- year [6]. In April 2014, the American Food and Drug Administration (FDA) approved cobas® 4800HPV testing as an option for primary screening, which provides genotyping information for HPV16/18, while simultaneously reporting the 12 other hrHPV types; therefore, women with potential positivity for the 12 other possible hrHPV genotypes are triaged by cytology [5]. Thus, whether adopting co-screening or HPV primary screening, there would be a proportion of HPV-positive women with normal cytology. On one hand, HPV testing has a low specificity and a low positive predictive value, which may increase colposcopy burden and overtreatment [7]. On the other hand, genotyping solely for HPV16/18 would miss a proportion of patients with high-grade cervical lesions, since mounting evidence suggests that the risk of high-grade squamous intraepithelial lesions, adenocarcinoma in situ and cervical cancer (HSIL+) in women positive for HPV31, 33, 52 and 58 is equivalent to or greater than that in women positive for HPV18 [8–10]. Therefore, managing hrHPV positive women with normal cytology is a major issue. Furthermore, HPV genotype prevalence and vaccination rates are diverse among regions. A meta-analysis reported that the most common HPV types detected in invasive cervical cancer (ICC) cases from Eastern Asia were HPV16, 18, 58, 52, 33, 31, 45 and 59 [11]. Another study showed that in cervical intraepithelial neoplasia (CIN) 2/3 and adenocarcinoma in situ samples, HPV 16, 52, 58, 51, 33, 31, 18 and 35 were the most common HPV types in five Asian countries [12]. The data collected from other countries may not, therefore, represent the situation in China [13, 14].

Introducing HPV testing for cervical cancer screening is becoming increasingly popular in China. Co-screening, HPV primary screening or cytology primary screening has been used in different districts of China due to unbalanced economic development. It is uncertain which combinations of hrHPV genotyping could provide the optimal triage of HPV-positive women with normal cytology in clinical practice in northeastern China. To address these concerns, a retrospective study was conducted to evaluate the prevalence of HPV, and the association between hrHPV genotypes and the detection of histological HSIL+ in northeastern China. Furthermore, an acceptable triage strategy to reduce the burden of cytological examination and increase the proportion of detecting histological HSIL+ was investigated.

## Methods

### Study population

We recruited women between 25 and 65 years old who underwent co-screening for cervical cancer when visiting outpatient of the Department of Obstetrics and Gynecology at the China Medical University-affiliated Shengjing Hospital between January 1st 2012 and December 31st 2014. The clinical characteristics and pathological data were obtained from the hospital’s electronic files, including age at diagnosis, cytology results, HPV genotyping results, colposcopy results and histological results. Exclusion criteria for the present study were pregnant women; women who had a hysterectomy; women with a history of cervical cancer; and women who had received previous treatment for any cervical epithelial lesion. The study was approved by the ethics committee of Liaoning Cancer Hospital and Institute (20190971).

### Cytology

Cytological testing was performed using ThinPrep® liquid-based cytology (Hologic Inc., MA, USA). Cell samples were collected using a cytobrush and placed into a tube with transport medium. The first sample from each woman was used for cytology analysis. The second sample was taken for HPV genotyping testing. Slides were screened by two cytotechnologists and diagnosed according to the 2001 Bethesda system.

### HPV genotyping

HPV genotyping was identified by the HPV GenoArray test kit (Hybribio Ltd., Hong Kong). This assay was performed using both DNA amplification by the L1 consensus primer-based polymerase chain reaction (PCR) and a flow-through hybridization technique. A total of 21 genotypes were screened, including13 high-risk genotypes (HPV16, HPV18, HPV31, HPV33, HPV35, HPV39, HPV45, HPV51, HPV52, HPV56, HPV58, HPV59 and HPV68), two probable high-risk genotypes (HPV53 and HPV66) and six low-risk genotypes (HPV6, HPV11, HPV42, HPV43, HPV44 and CP8304) [15]. Positive and negative control samples were included in each experiment.

### Colposcopy and biopsy

All women with abnormal cytology or those who tested positive for HPV were referred for colposcopy. A colposcopy-guided biopsy was performed if a suspicious lesion was found. Random cervical biopsy was carried out when colposcopic inspection was inadequate. If colposcopy results were normal upon adequate inspection, women were followed up yearly without biopsy. The grade of the cervical lesion was independently diagnosed by two expert pathologists, according to the 4th edition of the WHO Women’s Reproductive System Tumor Classification [16]. Histological high-grade squamous intraepithelial lesion (HSIL) included CIN2, CIN3 and squamocarcinoma in situ. The suffix “+” meant the indicated or more severe histology. HSIL, adenocarcinoma in situ and cervical cancer were designated as HSIL+ in the present study. Immunohistochemical stains for p16 and Ki67 were used when a consensus was not reached.

### Data analysis

The prevalence of HPV was expressed as a proportion of the number of HPV-positive cases compared to the total number of cases tested for HPV. Multiple HPV infections were defined as those positive for two or more types of HPV. Women with multiple HPV infections were counted more than once for each positive genotype. The prevalence of specific HPV types is presented for the 13 hrHPV types and two probable hrHPV types in women with hrHPV infection, as well as hrHPV positive women with normal cytology and histological HSIL+ women who received colposcopy with hrHPV positivity and normal cytology. Chi-squared (χ^2^) tests were used to compare differences of the proportions of histological HSIL+ in hrHPV positive women with normal cytology who received colposcopy, across all four age groups, and among hrHPV genotypes, as well as between each pairing of two genotypes. Logistic regression analysis, which was adjusted for age, was used to estimate the association between specific hrHPV genotypes and the proportion of detecting histological HSIL+. Odds ratios (ORs) with 95% confidence intervals (CIs) were calculated. Data were analyzed using the SPSS version 22.0 software (SPSS Inc., Chicago, IL, USA). A *P* value of < 0.05 was considered statistically significant.

## Results

### Characteristics of the study population

**Fig. 1:**
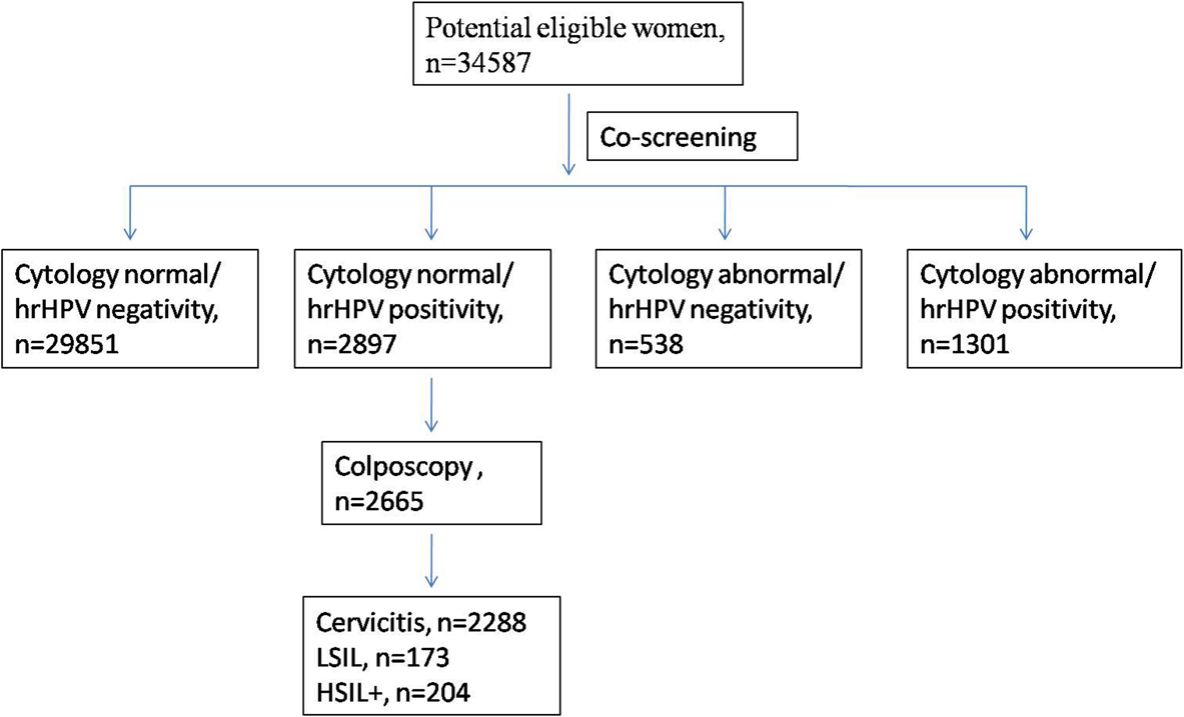
Flowchart for the study population. (hrHPV, high risk human papillomavirus; HSIL+, histologic high-grade squamous intraepithelial lesions, adenocarcinoma in situ and cervical cancer)

A total of 34,587 women aged 25–65 years old attended our hospital for cervical cancer co-screening; 4198 of these women (12.1%) had hrHPV infection and 1839 (5.3%) had abnormal cytology results (Fig. 1). Multiple HPV infections were detected in 676 of the 4198 (16.1%) infected women. The most common hrHPV genotype was HPV16 (1373, 32.7%), followed by HPV58 (680, 16.2%), HPV52 (571, 13.6%), HPV53 (504, 12.0%), HPV33 (360, 8.6%) and HPV18 (301, 7.2%).

### hrHPV positive women with normal cytology

Of the 4198 hrHPV infected women, 2897 (69.0%) women with normal cytology were analyzed in the present study. The mean age of these women was 39.60 ± 8.99 years old, with a median age of 40. The top six hrHPV genotypes were HPV16 (874, 30.2%), HPV58 (452, 15.6%), HPV52 (395, 13.6%), HPV53 (380, 13.1%), HPV18 (224, 7.7%) and HPV33 (208, 7.2%). Regarding each hrHPV genotype, the proportion of women with normal cytology was higher in those infected with HPV59 (76.3%), HPV45 (75.8%), HPV53 (75.4%), HPV18 (74.4%) and HPV51 (70.65%) than other hrHPV genotypes (Table 1).

**Table 1:** The prevalence of hrHPV genotypes in 2897 hrHPV positive women with normal cytology

hrHPV genotypes	Total, *n* = 4198, (%)	No. of hrHPV positive women with normal cytology *n* = 2897, (%)	The percentages of women with normal cytology in hrHPV positive women (%)	No. of histological HSIL+, *n* = 204, (%)	No. of histological high-grade squamous intraepithelial lesion, *n* = 173, (%)	No. of histological adenocarcinoma in situ and adenocarcinoma, *n* = 7, (%)	No. of histological squamous cervical cancer, *n* = 24, (%)
16	1373(32.7)	874(30.2)	63.7	119(58.3)	99(57.2)	2(28.6)	18(75.0)
18	301(7.2)	224(7.7)	74.4	12(5.9)	6(3.5)	5(71.4)	1(4.2)
31	221(5.3)	154(5.3)	69.7	15(7.4)	13(7.5)	0	2(8.3)
33	360(8.6)	208(7.2)	57.8	22(10.8)	21(12.1)	1(14.3)	0
35	41(1.0)	22(0.8)	53.7	0	0	0	0
39	234(5.6)	169(5.8)	72.2	5(2.5)	5(2.9)	0	0
45	62(1.5)	47(1.6)	75.8	2(0.1)	2(1.1)	0	0
51	143(3.4)	101(3.5)	70.6	4(2.0)	3(1.7)	0	1(4.2)
52	571(13.6)	395(13.6)	69.2	28(13.7)	28(16.2)	0	0(0)
53	504(12.0)	380(13.1)	75.4	10(4.9)	9(5.2)	0	1(4.2)
56	75(1.8)	37(1.3)	49.3	0	0	0	0
58	680(16.2)	452(15.6)	66.5	26(12.7)	25(14.5)	0	1(4.2)
59	93(2.2)	71(2.5)	76.3	1(0.05)	0	0	1(4.2)
66	209(5.0)	143(4.9)	68.4	7(3.4)	7(4.0)	0	0
68	166(4.0)	115(4.0)	69.3	4(2.0)	4(2.3)	0	0

Women with multiple HPV types detected are counted to each type, and therefore counted more than once

### hrHPV genotype and HSIL+ incidence

Colposcopy was performed in 2665 HPV-positive women with normal cytology. Colposcopy-guided biopsy and random biopsy were carried out in 1742 (1742/2665, 65.4%) women. A total of 204 women (204/2665, 7.7%) had HSIL+; 173 women (173/2665, 6.5%) had low-grade squamous intraepithelial lesions; and 2288 women (2288/2665, 85.8%) had negative histology results or normal colposcopy results. Of the 204 women with HSIL+, 26 women (26/204, 12.7%) had ICC (24 cases with squamous cervical cancer and two cases with adenocarcinoma), including 14 women (14/26, 53.8%) who were FIGO stage IA. In the HSIL+ group, the prevalence of the hrHPV genotypes was HPV16 (119, 58.3%), HPV52 (28, 13.7%), HPV58 (26, 12.7%), HPV33 (22, 10.8%), HPV31(15, 7.4%) and HPV18 (12, 5.9%). The percentage of women infected with multiple hrHPV genotypes was 19.1% (39/204) in the HSIL+ group. In the squamous cervical cancer group, the prevalence of HPV16 (18/24, 75%) was prominent. Of the seven cases of adenocarcinoma in situ and adenocarcinoma, five cases were HPV18-positive and two cases were HPV16-positive, including one case of dual infection with HPV18 and HPV33 (Table 1).

### hrHPV genotypes and detection of histological HSIL+

The proportion of histological HSIL+ in hrHPV positive women with normal cytology who received colposcopy did not differ significantly by age group (divided into 25–34, 35–44, 45–54 and 55–65 years old. *P* = 0.42). Of the 801 HPV16-positive women with normal cytology who received colposcopy, 119 (119/801, 14.9%) women were confirmed as HSIL+ by histological diagnosis. The proportion of histological HSIL+ in each genotype was greater than10% for HPV16 (14.9%), HPV33 (22/187, 11.8%) and HPV31 (15/142, 10.6%). The proportion of histological HSIL+ in each genotype was less than 10% for HPV52 (28/362, 7.7%), HPV58 (26/414, 6.3%), HPV18 (12/206, 5.8%) and other types. Compared to women infected with HPV16, the proportion of histological HSIL+ was not significantly different in women infected with HPV33 or HPV31 (*P* = 0.30, *P* = 0.19, respectively). While HPV58, HPV52, HPV53 and HPV18 were four of the top six most common genotypes in HPV-positive women with normal cytology, the proportion of histological HSIL+ in each of these four HPV genotypes was significantly lower than those in women infected with HPV16 (*P* < 0.001, *P* = 0.001, *P* < 0.001 and *P* < 0.001, respectively). The proportion of histological HSIL+ in women infected with HPV33 was significantly higher compared to those infected with HPV18 or HPV58 (*P* = 0.047, *P* = 0.03, respectively). The differences between the proportion of histological HSIL+ in women infected with HPV18 and those infected with the HPV31, HPV52 or HPV58 were not statistically significant (*P* = 0.15, *P* = 0.50, *P* = 1.00, respectively) (Table 2). The proportion of histological HSIL+ in women infected with multiple genotypes was significantly higher than those with a single infection (*P* = 0.03) (Table 2).

**Table 2:** Comparison of the proportions of HSIL+ among different age groups and hrHPV genotypes in hrHPV positive women with normal cytology who received colposcopy

		No. of hrHPV positive women with normal cytology who received colposcopy, *n* = 2665	No. of histological HSIL+	Percentages of HSIL+	χ^2^ value	*P* value		
Age groups	25–34	923	61	6.6	2.81	0.42		
	35–44	975	84	8.6				
	45–54	585	46	7.9				
	55–65	182	13	7.1				
HPV infection	single	2296	165	7.2	5.15	0.03		
	multiple	369	39	10.6				
hrHPV genotype	16	801	119	14.9	82.37*	< 0.001*		
	18	206	12	5.8		< 0.001^a^		
	31	142	15	10.6		0.19^a^	0.15^b^	0.86^c^
	33	187	22	11.8		0.30^a^	0.047^b^	
	35	21	0	0				
	39	155	5	3.2				
	45	43	2	4.7				
	51	93	4	4.3				
	52	362	28	7.7		0.001^a^	0.50^b^	0.19^c^
	53	349	10	2.9		< 0.001^a^		
	56	34	0	0				
	58	414	26	6.3		< 0.001^a^	1.00^b^	0.03^c^
	59	65	1	1.5				
	66	131	7	5.3				
	68	105	4	3.8				

Note *among hrHPV genotypes, ^a^compared to HPV16, ^b^compared to HPV18, ^c^compared to HPV33

Among all 2665 women with hrHPV-positive and cytology-negative results, following adjustment for age, the odds ratio (OR) for histological HSIL+ was 3.75 (95% CI = 2.79–5.05) in women with HPV16 infection, compared to women with non-HPV16 infection. In women infected with HPV33, the OR for histological HSIL+ was 1.69 (95% CI = 1.04–2.72). Infection with HPV genotypes 18, 31, 52 or 58 did not increase the risk of HSIL+ (OR = 0.72, 1.46, 1.03, 0.78, 95% CI = 0.39–1.32, 0.83–2.57, 0.68–1.57, 0.51–1.20, respectively). The OR for histological HSIL+ was 3.26 (95% CI = 2.41–4.40) in women with HPV16/18 infection. The OR for histological HSIL+ was 4.21 (95% CI = 2.99–5.93) in women infected with HPV16/18/31/33. The OR for histological HSIL+ was 5.73 (95% CI = 3.30–9.97) in women infected with HPV16/18/31/33/52/58 (Table 3).

**Table 3:** Detection of histologic high-grade squamous intraepithelial lesion or worse lesions by different hrHPV genotyping approaches in the study population

	No. of hrHPV positive women with normal cytology and with availble histologic or colposcopic results	No. of histologic HSIL+	Percentage of HSIL+ detected, n = 204	Ratio of colposcopy per HSIL+ detection	OR	95% CI	*P* value
HPV16	801	119	58.3	6.7	3.75	2.79–5.05	< 0.001
HPV18	206	12	5.9	17.2	0.72	0.39–1.32	0.29
HPV31	142	15	7.4	9.5	1.46	0.83–2.57	0.19
HPV33	187	22	10.8	8.5	1.69	1.04–2.72	0.03
HPV52	362	28	13.7	12.9	1.03	0.68–1.57	0.90
HPV58	414	26	12.7	15.9	0.78	0.51–1.20	0.26
HPV16/18	992	129	63.2	7.7	3.26	2.41–4.40	< 0.001
HPV16/18/33	1162	148	72.5	7.9	3.85	2.79–5.31	< 0.001
HPV16/18/31/33	1282	158	77.5	8.1	4.21	2.99–5.93	< 0.001
HPV16/18/31/33/52/58	1938	190	93.1	10.2	5.73	3.30–9.97	< 0.001

HPV16/18 infection was detected in 129 of 204 (63.2%) women with histological HSIL+; by contrast, the top six hrHPV genotypes (HPV16/18/31/33/52/58) in the HSIL+ group were detected in 190 (190/204, 93.1%) women. However, the colposcopy per HSIL+ detection ratio also increased significantly from 7.7 to 10.2 (*P* = 0.01). Adding the HPV31/33 genotype to the HPV16/18 genotype increased the percentage of HSIL+ detection from 63.2 to 77.5% (*P* = 0.002) without significantly increasing the colposcopy per HSIL+ detection ratio (7.7 to 8.1, *P* = 0.66). (Table 3).

## Discussion

Cervical cancer screening has regional differences in China. In relatively developed areas of China, co-screening is commonly performed in hospitals [10], and it is clear that women with abnormal cytology and hrHPV positivity should be referred for colposcopy [17]; however, the management of hrHPV positive women with normal cytology remains controversial. The triage of HPV primary screening faces the same problem. Several studies have shown that the current cervical screening strategy with HPV16/18 genotyping misses some non-HPV16/18 infected women who progress to high-grade cervical lesions or cancer [18, 19]. The present study was a real-world study and evaluated the prevalence of hrHPV genotypes and the correlation with HSIL+ risk, especially in hrHPV positive women with normal cytology.

The prevalence of hrHPV (12.1%) obtained in the present study was lower than that reported in many Chinese cities [20]; however, it was slightly higher than that (9.5%) reported in a previous study from the same region [21]. Moreover, previous population-based screening results have demonstrated that the overall prevalence of hrHPV varies from 9.9–27.5% across China [22]. A previous study suggested possible reasons for this inconsistency, such as, different study populations, geographical prevalence, and differences in detection methods [23]. The HPV GenoArray test was a PCR-based kit that detected individual HPV genotypes. This technology has not been approved by the FDA, which could be a limitation of the study. Several studies have shown that the HPV GenoArray test is a reliable method for detecting and genotyping HPV infections [15, 24]. In accordance with previous data reported by Chinese population-based investigations [20–22], HPV16, HPV58 and HPV52 were found to be the dominant hrHPV types in the present study, followed by HPV53, HPV33 and HPV18. However, the results were distinctly different from those reported by a summarized global meta-analysis, in which HPV16 was the most frequently detected type; HPV18 ranked second place in CIN3 and ICC; HPV45 was more common than other non-HPV16/18 types in ICC [25]. In the present study, the most common genotypes in hrHPV positive women with normal cytology were mostly in accordance with those in all hrHPV positive women, with the exception that HPV18 was moved up to fifth place and HPV33 was moved down to sixth place.

The oncogenic potential varies with different hrHPV genotypes. A population-based study showed that HPV16, 58, 18, 52 and 33 were most common in persistent infection [26]. Another study showed that HPV16, 33 and 58 increased the risk of HSIL+ as compared with hrHPV-negative women [27]. Moreover, it has been shown -by Bayesian probability modeling – that there is the highest risk of HSIL+ in HPV16-positive patients; furthermore, HPV31- and HPV33/58-positive patients have a higher risk of HSIL+ compared to HPV18-positive patients [28]. A European study showed that the most common HPV types in women with HSIL and cervical cancer were HPV16/33/31 (59.9/10.5/9.0%) and HPV16/18/45 (63.3/15.2/5.3%), respectively [29]. In a study from Denmark, HPV16, HPV18, HPV31 and HPV33 infection and especially HPV16 persistence were associated with high absolute risks for progression to high-grade cervical lesions [30]. In the present study, in women with normal cytology, HPV16 was the most common genotype in histological HSIL+; moreover, HPV52, 58, 33 and 31 were more common than HPV18. Moreover, the proportion of histological HSIL+ was not significantly different between women infected with HPV33 or HPV31 and women infected with HPV16. Although the prevalence of HPV53 was common, there was a low risk of developing HSIL+. In a study from Norway using a 5-type HPV mRNA test, after 6 years of follow-up, the cumulative proportions of high-grade cervical lesions were significantly higher in women who were HPV16 positive at baseline compared to women who were HPV31/33/45 positive at baseline. There were no differences, however, in high-grade cervical lesions between women who were HPV16 positive and women who were HPV18 positive at baseline [31]. Therefore, more studies are needed to confirm these results.

HPV genotyping will enable more precise characterization of cervical disease risk, but genotyping for only HPV16/18 is not sufficient. Although the prevalence and risk of HSIL+ in women with HPV18 didn’t rank high among non-HPV16 types, HPV18 was one of the most common genotypes in adenoepithelial lesions. In the present study, HPV16/18 was positive in 63.2% of women with histological HSIL+. The addition of HPV31/33 genotyping to that of HPV16/18 could detect 14.3% more women with histological HSIL+. The OR for histological HSIL+ in women infected with HPV16/18/31/33 was higher than that in women infected with HPV16/18 (4.21 vs. 3.26). The addition of HPV31/33/52/58 genotyping to that of HPV16/18 could detect 93.1% of histological HSIL+ in the present study. The OR for histological HSIL+ was 5.73 in women infected with HPV16/18/31/33/52/58. A previous population-based, prospective, observational study suggested that HPV16/18/31/33/52/58 infection should be immediately referred for colposcopy [32]. However, in the present study, women infected with HPV16/18/31/33/52/58 accounted for 72.7% (1938/2665) of all hrHPV positive women with normal cytology; accordingly, the burden of colposcopy would increase. The results from our study support the need for immediate colposcopy in women infected with HPV16/18/31/33 in order to detect more HSIL+ cases; in addition, the colposcopy burden did not increase significantly. If colposcopy resource is sufficient, it is also recommended that women with HPV16/18/31/33/52/58 infection have immediate colposcopy.

## Conclusions

In summary, wider hrHPV genotyping provides a better predictive value than HPV16/18 genotyping alone in guiding the clinical management of current cervical cancer screening. In northeastern China, the addition of HPV31/33 genotyping to that of HPV16/18 should be recommended in triaging women with a positive HPV test.

## Data Availability

The dataset analyzed in this study is available from the corresponding author upon reasonable request.

## Abbreviations

HPV: Human papillomavirus
hrHPV: high-risk human papillomavirus
ACS: American Cancer Society
ASCCP: American Society for Colposcopy and Cervical Pathology
ASCP: American Society for Clinical Pathology
FDA: the American Food and Drug Administration
ICC: Invasive cervical cancer
CIN: Cervical intraepithelial neoplasia
HSIL: High-grade squamous intraepithelial lesion
PCR: Polymerase chain reaction
OR: Odds ratio
CI: Confidence interval
FIGO: Federation International of Gynecology and Obstetrics
MA: Massachusetts
USA: United States of America
